# Outcome of CO2 laser vaporization for oral potentially malignant disorders treatment

**DOI:** 10.4317/medoral.21984

**Published:** 2017-02-25

**Authors:** Alexandra Cloitre, Rafael W. Rosa, Elise Arrive, Jean-Christophe Fricain

**Affiliations:** 1 DDS; Inserm UMR 1229, RMeS, Regenerative Medicine and Skeleton, Université de Nantes, ONIRIS, Nantes, F-44042, France; Université de Nantes, UFR Odontologie, Nantes, F-44042, France; CHU Nantes, Service Odontologie Restauratrice et Chirurgicale, PHU4 OTONN, Nantes, F-44093, France; 2 DDS, MPH; ISPED, Université de Bordeaux, Bordeaux, F-33076, France; 3 DDS, PhD; ISPED, Inserm U 1219, Bordeaux Population Health Research Center, Bordeaux, F-33076, France; Université de Bordeaux, UFR des Sciences Odontologiques, Bordeaux, F-33082, France; CHU Bordeaux, Pôle d’Odontologie et de Santé Buccale, Bordeaux, F-33000, France; 4 DDS, PhD, HDR; Inserm U1026, BioTis, Bioingénierie Tissulaire, Université de Bordeaux, Bordeaux, F-33076, France; Université de Bordeaux, UFR des Sciences Odontologiques, Bordeaux, F-33082, France; CHU Bordeaux, Pôle d’Odontologie et de Santé Buccale, Bordeaux, F-33000, France

## Abstract

**Background:**

Oral cancer is a public health issue worldwide. Oral potentially malignant disorders (OMPDs) are lesions of the oral mucosa that are predisposed to malignant transformation. The mainstay of OMPDs treatment around the world is now the carbon dioxide (CO2) laser but the reported recurrence and malignant transformation rates vary widely in the literature. We aimed to estimate the recurrence and the malignant transformation rates of OPMDs treated with CO2 laser at the University Hospital of Bordeaux, in France, from 2010 to 2014, and to identify associated factors with recurrence or malignant transformation.

**Material and Methods:**

We conducted a retrospective study in patients with a minimum follow-up of 12 months. Collected variables included characteristics of the patients (gender, age, alcohol and tobacco consumption, previous diagnosis of graft-versus-host disease, previous treatments for OPMD or for upper aerodigestive tract cancers and human immunodeficiency virus infection), characteristics of the lesions (form, colour, size, location, degree of dysplasia), laser treatment outcome (complications, recurrence, malignant transformation).

**Results:**

Twenty-five patients were included. Mean follow-up was 28.9 months. Recurrence was observed in 11 patients (44%). Annual recurrence rate was 18.3% and annual malignant transformation rate was 1.7%. Hyperplasia without dysplasia was the only factor found to be statistically associated with recurrence.

**Conclusions:**

Our results suggest that OMPDs treated by CO2 laser vaporization have high recurrence rates, particularly those presenting hyperplasia. A standardized definition of recurrence would be necessary for inter-study comparisons. Long-term follow-up is recommended in order to detect and treat squamous cell carcinoma in its early stages.

** Key words:**CO2 lasers, precancerous conditions, malignant transformation, oral cancer, recurrence.

## Introduction

Cancer is becoming the first cause of premature deaths among noncommunicable diseases according the World Health Organization (WHO). An estimated 300 373 new cases of cancers of the lip and oral cavity occurred worldwide in 2012, with 145 353 deaths (WHO). Most oral cancers are oral squamous cell carcinoma. The main risk factors for oral cancer are tobacco smoking and chewing and alcohol consumption. Prognosis of the oral cancer decreases with advanced disease at presentation. The five-years survival rates are approximately 80% for stage I cancers, and 20% for advanced diseases (stages III/IV) (1). The treatment of oral cancer of early stage permits to improve survival and to reduce morbidity in patients (2). Unfortunately worldwide the half of patients present with a late stage of disease (1). Management of oral potentially malignant disorders (OMPDs) to prevent malignant transformation (primary prevention, not evidence-based to date (3)) or to detect very early cancer (secondary prevention) is worthy of consideration (2).

OMPDs are lesions of the oral mucosa that are predisposed to malignant transformation. The most common OMPD in France include leukoplakia (homogeneous leukoplakia, nonhomogeneous leukoplakia and proliferative verrucous leukoplakia (PVL)), lichen planus, lichenoid dysplasia and actinic cheilitis ( Table 1) (4-10). Standard care of these lesions are surgical removal of the lesion (cold-knife, laser excision and vaporization, cryosurgery, photodynamic therapy), medical treatment (topical or systemic), cessation of risk activities (smoking and alcohol) and surveillance (3). Carbon dioxide (CO2) is now the mainstay of treatment of OPMD around the world (11), it has been previously reported to be a safe and an effective tool (12-15). The reported recurrence and malignant transformation rates vary widely after treatment of OPMDs because of differences in terms of lesions treated, treatment, follow-up time and definition of recurrence, which leads to difficulties in informing patients (9-11,13,14,16-18).

**Table 1:**
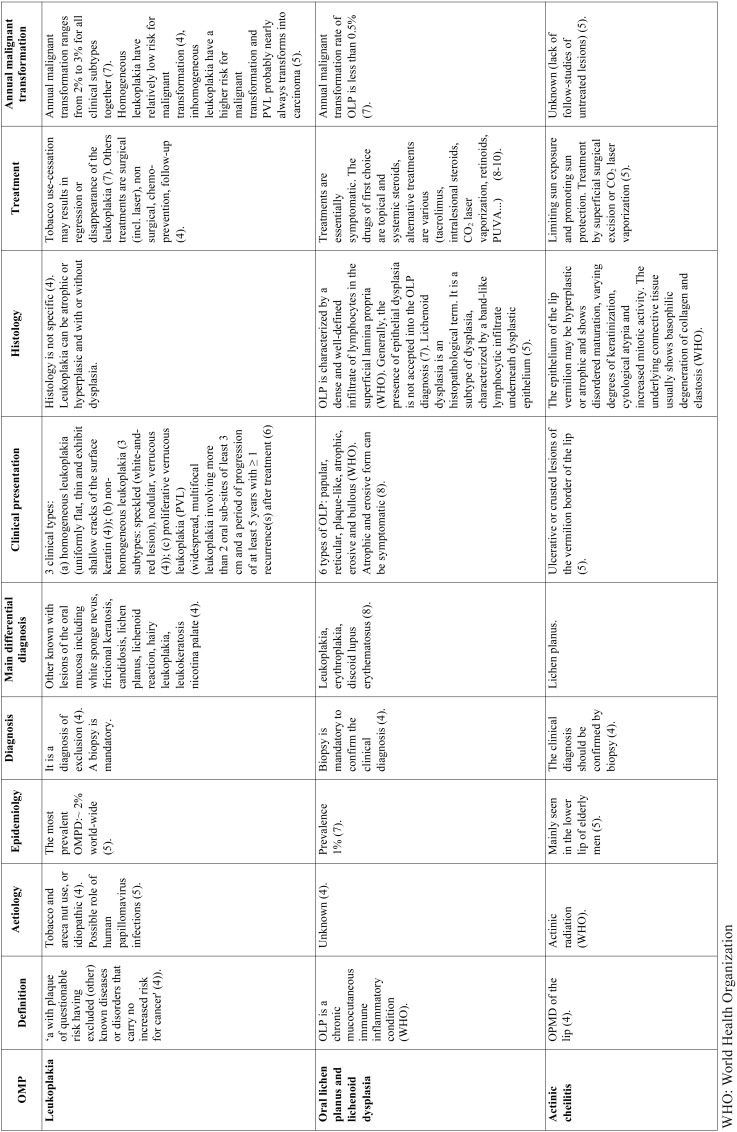
Literature review on the most common oral potentially malignant disorders (OMPDs) in France.

The objective of the present study was to estimate the recurrence and the malignant transformation rates of OPMDs treated with CO2 laser at the University Hospital of Bordeaux, in France, from 2010 to 2014, and to identify associated factors with recurrence or malignant transformation.

## Material and Methods

- Study design

This retrospective study included all patients treated with CO2 laser for oral potentially malignant disorders (OPMD) at the University Hospital of Bordeaux from 2010 to 2014 with a minimum follow-up of 12 months. Follow-up started from the date of first laser treatment and ended on the date of the last visit to the hospital in 2014 or on the date of malignant transformation. Written informed consent was obtained from each patient. The study was approved by the French ethics committee Comité de Protection des Personnes Sud-Ouest et Outre Mer III.

- Laser treatment

All included OPMDs were treated with laser vaporization after incisional biopsies. Lesions with severe dysplasia were not included because they were surgical excised to allow histological examination of the whole lesion. The CO2 laser system was a Lumenis ® 40C (Lumenis Inc., CA, USA). Laser wavelength was 10966 nm, the beam was used at focal spot 4, power from 10 to 20W, in a noncontact application. The same surgeon treated all patients. Treatment was performed under local anaesthesia (articaïne with epinephrine 1:100000). Lesions were delimited with a surgical marker with a 2-3 mm margin when feasible. Paracetamol, alone or combined with codeine, was prescribed for pain control after treatment.

- Data collection

• Patients: The characteristics of the patients treated with laser vaporization included the demographic cha-racteristics (gender, age), the risk factors (alcohol and tobacco consumption (user, non-user, or former user), previous diagnosis of graft-versus-host disease, previous treatments for OPMD or for upper aerodigestive tract cancers and human immunodeficiency virus infection (5)) and the follow-up (number of medical appointments and laser sessions).

• Oral potentially malignant disorders: The OMPD included were homogeneous leukoplakia, nonhomogeneous leukoplakia, proliferative verrucous leukoplakia (PVL), lichen planus, lichenoid dysplasia and actinic cheilitis ( Table 1). The lesions were described according to their form (unifocal, multiple sites, symmetric multiple sites), colour (white, red, black), size of greatest dimension (under 2 cm, from 2 to 4 cm, over 4 cm), and location (tongue, upper lip, lower lip, oral commissures, upper alveolus and gingiva, lower alveolus and gingiva, floor of mouth, buccal mucosa, hard palate and soft palate). The degree of dysplasia was classified as hyperplasia without dysplasia, or with dysplasia (mild, moderate).

• Laser treatment outcome.

◦ Complications: All complications after laser treatment such as necrosis of the treated area, granuloma onset were taken into account.

◦ Recurrence: Local recurrence was defined as an OMPD arising within the borders of the treated area after complete remission. Small white lesions arising between laser sessions were not considered as recurrence. Recurrence time was the period in months from the first laser session until the appearance of a recurrent OMPD. ◦ Annual recurrence rate was calculated as (15,16):

◦ Malignant transformation: Malignant transformation was defined as the development of oral squamous cell carcinoma or carcinoma in situ at the site of a previously treated OPMD. Malignant transformation time was the period in months from the first laser session until the development of oral squamous cell carcinoma or carcinoma in situ. Annual malignant transformation rate was calculated with the same formula using the total number of malignant transformations instead of the total number of recurrences.

◦ Clinical outcome categories: At the time of data extraction, patients were assigned to one of the 4 clinical outcome categories: complete remission (the absence of OPMD), under treatment or scheduled for treatment (achieving treatment or additional treatment required), stable on surveillance (OPMD persisting despite treatment) and malignant transformation (confirmed histologically).

- Data extraction: Data were extracted from medical records. Patients with missing risk factor data were contacted and asked by phone. When available, photographs were used to check the clinical characteristics of the lesions reported on medical records.

• Data analysis

Frequencies and percentages were calculated for qualitative variables. Mean, standard deviation (SD), and minimum (min) and maximum (max) were calculated for quantitative variables. The log-rank test and Kaplan-Meier survival curves were used to compare the 2 groups of patients (with recurrence or malignant transformation and without recurrence or malignant transformation) regarding the potential risk factors for recurrence: gender, age (in 2 categories, under 65 and 65 or over), tobacco and alcohol use, previous treatment, graft-versus-host disease and human immunodeficiency virus infection, lesion shape, colour, size, location, clinical and pathological diagnosis (mild and moderate dysplasia cases were grouped together). Tests were considered statistically significant if the *p*-value was <0.05. RStudio 0.98.1062 (RStudio, Inc.) was used for tests and description of variables.

## Results

1/ Patients

Of 46 patients examined for an OMPD and treated with laser vaporization from 2010 to 2014 at the University Hospital of Bordeaux, 21 were excluded because they did not have at least 12 months of follow-up at the time of data extraction (September 2014) (15,16). The remaining 25 patients were included: 14 men and 11 women, mean age 66.4 years (SD 11.1, min 49, max 90) ( Table 2). Seventeen patients (68.0%) reported a tobacco use (user and former user), 10 (43.5%) an alcohol use, and 3 (14.3%) a tobacco and alcohol use. Previous treatments for OPMD or for upper aerodigestive tract cancers were found in 5 cases (20.0%), GVH and HIV in 2 cases (8.0%). Follow-up ranged from 12.3 to 50.7 months (mean 28.9, SD 12.6). The mean number of medical appointments per patient was 8.3 (SD 3.5, min 2, max 17). The mean number of laser sessions was 4 (SD 2.2, min 1, max 9).

**Table 2:**
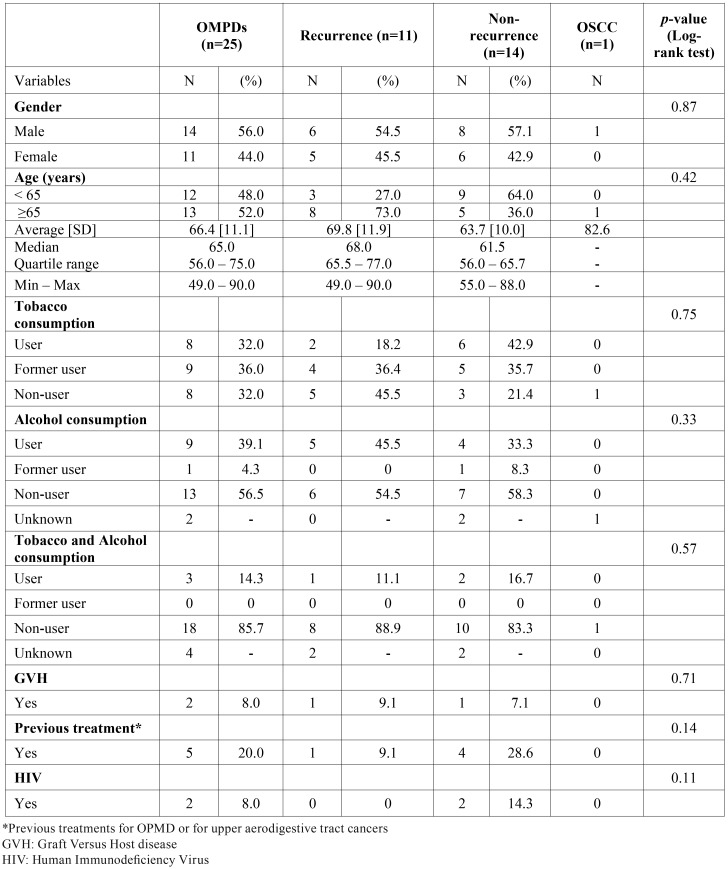
Characteristics of patients diagnosed with oral potentially malignant disorders (OPMDs), recurrent OPMDs and oral squamous cell carcinoma (OSCC). Centre Hospitalier Universitaire de Bordeaux, France, 2010-2014.

2/ Oral potentially malignant disorders

These 25 patients presented 25 OMPDs. Lesions were mainly unifocal (n=15, 60.0%), white (n=18, 75.0%), from 2 to 4 cm (n=9, 40,9%), at multiple site (n=10, 40%). 6 clinical diagnosis were encountered: homogeneous leukoplakia (n=11, 44.0%), nonhomogeneous leukoplakia (n=3, 12.0%), proliferative verrucous leukoplakia (PVL) (n=5, 20.0%), actinic cheilitis (n=1, 4.0%), lichen planus (n=3, 12.0%) and lichenoid dysplasia (n=2, 8.0%) ( Table 3). For the anatomopathologic diagnosis, 11 lesions were hyperplasia without dysplasia (44.0%) and 14 were with mild or moderate dysplasia (56.0%).

**Table 3:**
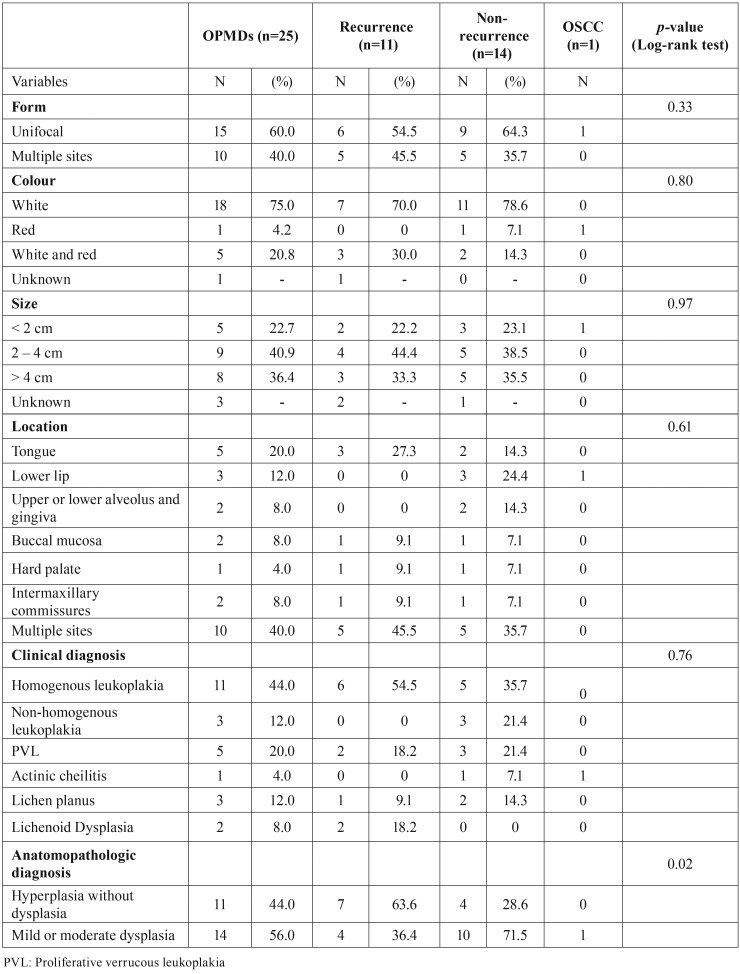
Characteristics of oral potentially malignant disorders (OPMDs), recurrences and oral squamous cell carcinomas (OSCC) diagnosed and treated among 25 patients. Centre Hospitalier Universitaire de Bordeaux, France, 2010-2014.

3/ Laser treatment outcome

**Figure 1:**
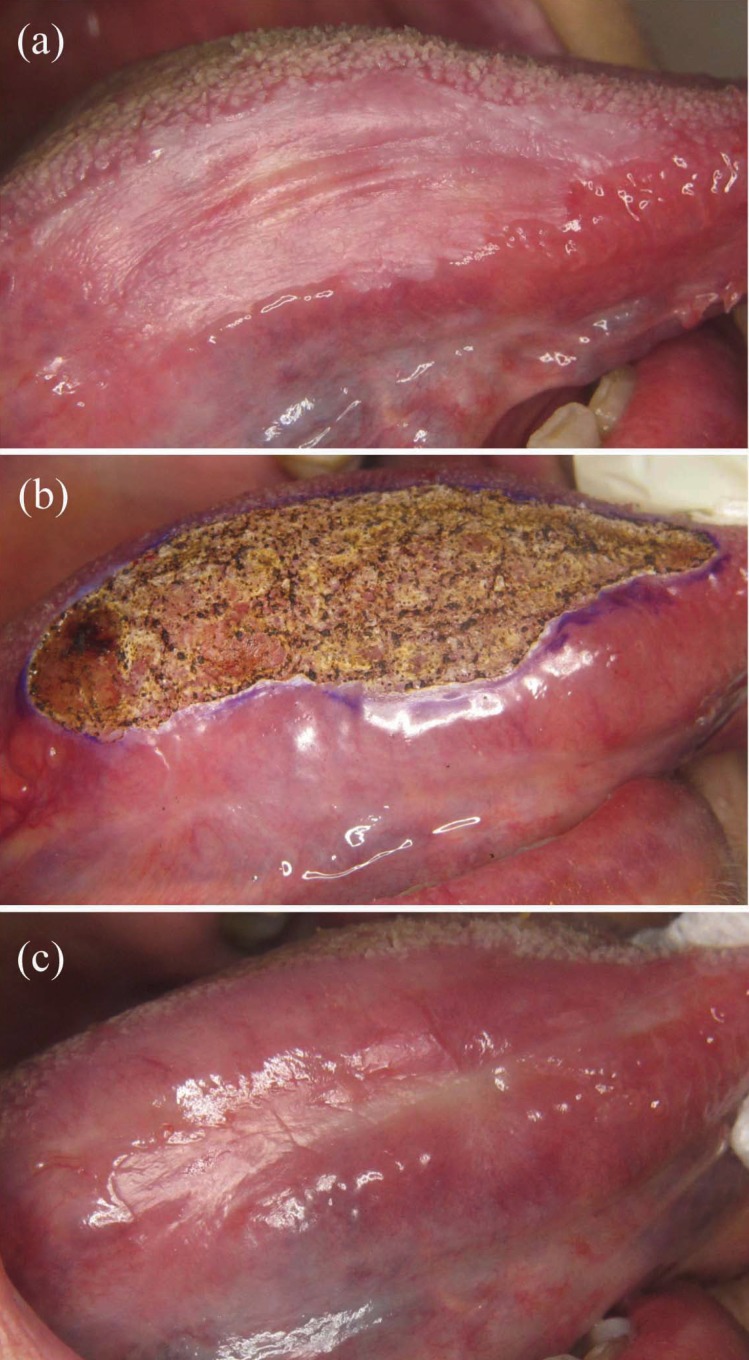
(a) Homogeneous leukoplakia on the tongue mucosa of a 77-year-old female patient. (b) Treatment by CO2 laser vaporization. (c) Persistent lesion 4 months after laser vaporization.

• Complications: Nine patients had minor complications (36%), 5 had granulomas (20.0%), 2 had small bone necrosis which resolved spontaneously (8.0%), 1 had delayed wound healing (4.0%) and 1 had a sclerotic scar (4.0%).

• Recurrence: Recurrence after laser treatment was observed in 11 of the 25 patients (44.0%). All recurrences appeared between 5.4 and 39.7 months after the first laser session (mean 19.7, SD 11.1; Fig. 1). Annual recurrence rate was 18.3%. Table 3 shows that most recurrent lesions were unifocal, 70.0% were white, 44.4% were 2-4 cm in size, 45.5% were located at multiple sites, and 54.5% were homogeneous leukoplakias. More than half of recurrent lesions initially had no dysplasia. Recurrence was managed by surveillance in 5 cases and by CO2 laser excision or vaporization in 6.

• Malignant transformation: In 1 of 25 treated patients (4%), malignant transformation occurred during the 48th month after the first laser treatment session. Annual malignant transformation rate was 1.7%. The patient with an initial diagnosis of actinic cheilitis and moderate dysplasia was later diagnosed with carcinoma in situ and was referred to the oncology department, where he is currently undergoing treatment

**Figure 2:**
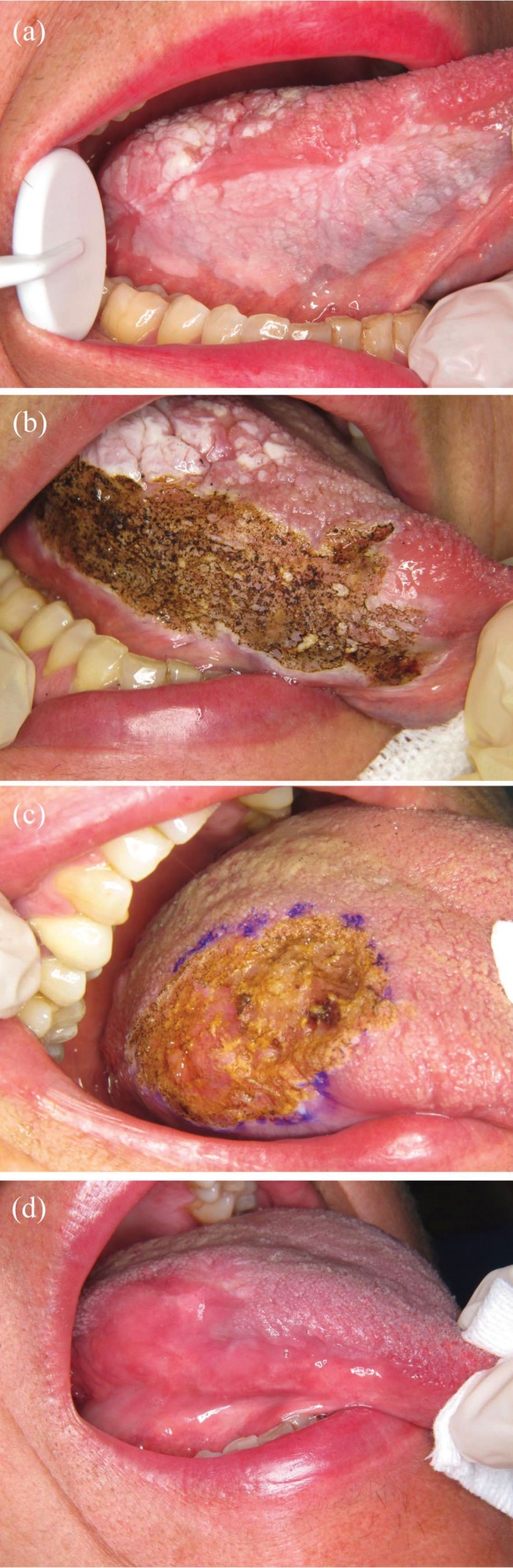
(a) Non-homogeneous leukoplakia on the tongue mucosa of a 57-year-old female patient. (b-c) Treatment by CO2 laser vaporization. (d) Complete remission 1 year after laser vaporization.

Clinical outcome categories: Excluding the lesion with malignant transformation, at the time of time of data extraction, 13 lesions (52%), including 4 that recurred, reached complete remissions (Fig. 2), 7 (28%) were stable on surveillance and 4 (16%) were under treatment or scheduled for treatment.

4/ Factors associated with recurrence or malignant transformation

Hyperplasia without dysplasia was the only factor found to be statistically associated with recurrence (*p*-value=0.02;  Table 3). No other factor/characteristic was found to be statistically associated with recurrence/malignant transformation ( Table 2 and  Table 3).

## Discussion

We considered all OPMDs diagnosed except those with severe epithelial dysplasia which were surgically excised (16). In the other lesions, recurrence after laser treatment occurred in 44% of the patients during a mean follow-up of mean 28.9 months. Our data showed a high rate of recurrence which was similar to that reported in some studies with CO2 laser vaporization ( Table 4): Brouns *et al.* reported a recurrence rate of 40% for oral leukoplakias in a mean period of 61.9 months (16), Pedrosa *et al.* observed recurrence rates of 40.7% for oral leukoplakias in a mean period of 43.8 months (17) and Mücke *et al.* reported a recurrence frequency of 38.2% for erosive lichen planus in a mean period of 42.7 months (9). In fact, the recurrence rate varies widely for CO2 laser vaporization from 9.9% to 44% (9-11,13,14,16-20);  Table 4). This variation can be attributed to the differences in terms of lesions treated, treatment modalities, follow-up time and above all definition of recurrence. There is no consensual definition for recurrence in the literature. Some authors define it as a lesion occurring at the primary lesion site after a confirmed remission period (17). Others note that only one-third of recurrences occur at the same site and consequently define recurrence as being a new lesion recurring after excision that possibly differs from the initial one (12). This definition favours the use of the term “disorder” instead of lesion, especially for leukoplakia, recognizing the fact that recurrence (or malignant transformation) can occur elsewhere in the mouth or in the upper aerodigestive tract (7). Different definitions of recurrence are a barrier to a clear comparison between studies. Recurrence could also result from a concept of field changes or cancerization: genetic changes widespread in the oral mucosa, triggered by environmental factors (e.g. smoking and alcohol), leading to higher degrees of dysplasia and to development of cancer (21). This could explain the high recurrence rate observed in this study. The role of field cancerization is especially important in widespread lesions like PVL, as demons-trated by Bagán *et al.* (22). The surgical margins are more likely to be within an area of field change: as area is wider, with clinically normal epithelium, recurrence is more likely to occur.

**Table 4:**
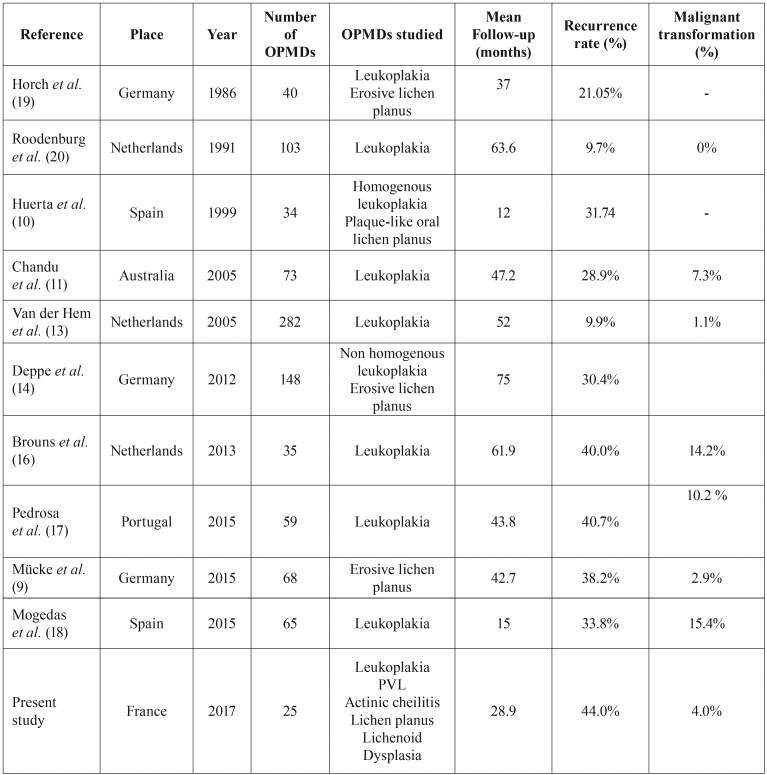
Literature review on the recurrence and malignant transformation rates of oral potentially malignant disorders (OMPDs) treated with CO2 laser vaporization.

We found no statistically significant association between exposure to patients’ characteristics and recurrence, except for the pathological diagnoses. Kaplan-Meier estimates indicated that, after 30 months of follow-up, the probability of being free from recurrence was approximately 75% for mild or moderate dysplasia lesions and about 50% for hyperplasia without dysplasia. In the literature, variables significantly associated with recurrence of OPMD treated with CO2 laser vaporization are alcohol consumption and previous malignancy (11), gingiva location (18) and moderate to high-grade dysplasia (17). Our contradictory findings can be explained by the fact that severe dysplasia was not included in the study (so the remaining dysplasia had a low risk of recurrence) and our small sample size. A greater sample size is needed to increase the chance of finding a significant association, but is difficult to reach in a single-centre study, as potentially malignant lesions are rare (<5%).

The ultimate aim of the management of OMPD is the prevention of oral cancer. To date, there is no evidence that a treatment for OPMD is effective for preventing the development of oral cancer (lack of Randomised Control Trial (RCT) with placebo or without treatment) (3). Holmstrup *et al.* reported in a retrospective study the long-term treatment outcome of 269 oral premalignant lesions (leukoplakia and erythroplakia) with or without surgical intervention by scalpel and they concluded that surgical intervention did not prevent malignant transformation (23). Although it yet remains unproven the surgical removal (including laser surgery) of the clinically altered tissue it is widely recommended (12). Our results indicate a malignant transformation rate of 4% in during a mean follow-up of 2.4 years (annual malignant transformation rate of 1.7%). It is in accordance with the malignant transformation rates of less than 15.4 % for OMPD treated with CO2 laser vaporization reported in the literature (9-11,13,14,16-20);  Table 4). Nevertheless, annual malignant transformation rates differ from one OPMD to another (2% to 3% for leukoplakia, less than 0.5% for lichen planus (7), unknow for actinic cheilitis (5), Table 1) and the diversity of the lesions included in our study making comparison difficult. A growing body of evidence suggests that effort should be made to differentiate oral lichen planus from oral lichenoid lesions, the latter showing a higher risk of malignant transformation. Differential diagnosis is considered hard (7). In this study we did not differentiate oral lichen planus and oral lichenoid lesions. We used the term ‘lichenoid dysplasia’ to determine lichenoid lesions presenting epithelial dysplasia, but this term is confusing (5,7) and more studies are needed to address the concept of lichenoid dysplasia in order to resolve any controversies related to the malignant potential of oral lichen planus.

The most relevant characteristics that increase the risk of malignant transformation are nonhomogeneous subtype, size (>4 cm), presence of dysplasia and location on the tongue or floor of the mouth (7). Advanced age and female sex are also important determinants in assessing malignant potential (24). In the present study, the association between these characteristics and malignant transformation was not tested because of the small number of cases. However, the literature shows that cessation of smoking after surgical treatment considerably reduces the risk of malignant transformation (7,25) and that patients should be encouraged to quit and referred to smoking cessation programs if necessary.

After CO2 laser vaporization, we noted minor complications in 9 cases (36%). Other researchers did not indicate the onset of complications (9,10,13,19), reported no complication (14,17,20) or significantly lower complication rates ranging from 2.9% to 7.7% (11,16,18). However, there is no standardized definition of a complication after treatment, so these findings may include different events. Exceptional cases of mental nerve paresthesia and anesthesia were reported (11,16).

Our study was not designed to evaluate the effect of the nature of the treatment. A comparative study with bilateral leukoplakia (one side of the lesion excised using CO2 laser and the other side using scalpel) has shown that advantages of laser include less bleeding intra operatively, less swelling and scarring post-operatively than scalpel (26). There was no significant difference in terms of post-operative pain. No RCT is available to compare CO2 laser with conventional surgery in terms of recurrence and malignant transformation rate. Brouns *et al.* 2014 have managed 144 patients with oral leukoplakia with surgical excision, CO2 laser vaporisation or observation only but was no unable to compare the treatment results due to their different indications (15). CO2 laser treatment modalities are vaporization i.e. a selective removal of affected epithelium, excision or a combination of both. The only comparative study available on laser evaporation and excision had used two different lasers for the treatment of leukoplakia (Nd:YAG and CO2 respectively) which did not allow to compare the laser treatment modalities (27). Laser vaporization appears suitable for wide lesions or multiple lesions at sites that were less amenable for surgical excision like floor of the mouth and mucobuccal folds (15,27). It permits to limit the post-operative discomfort and the functional problems induced by excision in this case (13,27). The main disadvantage is that vaporization does not allow histological examination of the whole lesion (13,27). Incisional biopsies performed prior to treatment may not represent the entire lesion and so some dysplasia may have been missed, resulting in a measurement bias (23). Besides risk factors, definition criteria and follow-up period, the type of laser and the surgical technique used may also be related to treatment outcome. RCT are needed to better establish the effectiveness of the different treatments for OPMD (3).

This study has several limitations. First, this is a retrospective study. It is based on complete records from the archives of the University Hospital of Bordeaux. Second, the sample size is small (25 patients) but similar to that reported in some studies with CO2 laser vaporization (10,16) ( Table 4). This is due to the characteristics of the studied lesions themselves which are rare (<5%) and require further follow-up to observe a potential recurrence or malignant transformation. We had a small sample size despite a four-year screening period, providing a small power to identify associated factors, but on the other hand, we had data of good quality from a homogeneous sample (severe dysplasia excluded, all treated by CO2 Laser by the same surgeon) followed-up at least 12 months as indicated by Brouns *et al.* (14,16). These lesions should become even rarer as the prevalence of tobacco smoking is declining worldwide (WHO), pointing out the need for future systematic reviews and meta-analyses, in which our study can contribute, and collaborative multicentre studies.

In conclusion, we noted a high recurrence rate of OMPDs treated with CO2 laser, particularly in those with hyperplasia. Randomized clinical trials should be used to evaluate the outcome of different treatment methods and multicentre studies could increase the power of statistical analyses. Standardized recurrence criteria are necessary for more accurate comparisons between studies. Long-term follow-up programmes are important to detect malignant transformation in its early stages and to minimize cancer treatment morbidity and mortality rates.
